# Evidence for Opportunity Cost Neglect in the Poor

**DOI:** 10.1002/bdm.2041

**Published:** 2017-09-11

**Authors:** Arnoud Plantinga, Job M.T. Krijnen, Marcel Zeelenberg, Seger M. Breugelmans

**Affiliations:** ^1^ Department of Social Psychology/TIBER Tilburg University Tilburg The Netherlands; ^2^ Anderson School of Management University of California Los Angeles CA USA; ^3^ Department of Marketing Vrije Universiteit Amsterdam Amsterdam The Netherlands

**Keywords:** opportunity costs, poverty, scarcity, judgment and decision making

## Abstract

People often neglect opportunity costs: They do not fully take into account forgone alternatives outside of a particular choice set. Several scholars have suggested that poor people should be more likely to spontaneously consider opportunity costs, because budget constraints should lead to an increased focus on trade‐offs. We did not find support for this hypothesis in five high‐powered experiments (total N = 2325). The experiments used different products (both material and experiential) with both high and low prices (from $8.50 to $249.99) and different methods of reminding participants of opportunity costs. High‐income and low‐income participants showed an equally strong decrease in willingness to buy when reminded of opportunity costs, implying that both the rich and the poor neglect opportunity costs. © 2017 The Authors Journal of Behavioral Decision Making Published by John Wiley & Sons Ltd.

Do the poor and the rich make financial decisions differently? Several studies show that they do. For example, the poor have been reported to discount the future more strongly (Green et al., 1996; Lawrance, 1991) and to be more risk averse (Dohmen et al., 2011). An important related question is whether the financial behavior displayed by the poor further contributes to a suboptimal financial position, leading to a vicious cycle of poverty (a poverty trap; Azariadis & Stachurski, 2005). Some research does suggest that this is the case; poverty was found to be related to decreases in cognitive functioning (Mani et al., 2013) and self‐control (Spears, 2011). However, there is also research suggesting that poverty can increase decision quality: The poor seem to be less susceptible to context effects and better able to judge the value of money (Shah et al., 2012). For example, in studies on the classic jacket and calculator problem, participants are usually willing to travel to a different store for a discount on a cheap product, but not for the same absolute discount on an expensive product (Tversky & Kahneman, 1981). However, in Shah et al., participants with lower incomes were not influenced by the price of the product, showing that they were less influenced by the decision context. Other studies find no differences between poor and rich in financial decision making. For example, Carvalho, Meier, and Wang (2016) found no differences in performance on cognitive tasks, heuristic judgements, or the consistency of intertemporal and risky choices between before‐payday and after‐payday groups. Bertrand, Mullainathan, and Shafir (2006, p. 8) argue that “the poor may exhibit basic weaknesses and biases that are similar to those of people from other walks of life, except that in poverty, there are narrow margins for error, and the same behaviors often manifest themselves in more pronounced ways and can lead to worse outcomes.” Taken together, these different findings strongly suggest that ideas about differences in financial decision making between the poor and the rich should not be taken at face value, but rather be empirically tested. In the current paper, we present five experiments testing whether poor and rich people differ in how they deal with opportunity costs.

Several scholars have predicted that the poor are less likely to suffer from opportunity cost neglect—failing to consider alternatives outside of a choice set which may result in suboptimal choices (Frederick et al., 2009; Jones et al., 1998; Legrenzi et al., 1993; Northcraft, 1986). In the words of Thaler (2015): “the one group of people that come closest to thinking this way [i.e. as described by normative theory] about opportunity costs is the poor [...] simply because opportunity costs are highly salient for them” (p. 58). Thinking about opportunity costs is important because money can only be spent once. The decision whether to buy something should not be based solely on a good's attributes, but also on potential alternative uses of people's money. Furthermore, opportunity costs should be especially important for the poor because their budget constraints leave only narrow margins of error (Bertrand et al., 2006); the same mistake can have more relative financial impact for the poor than for the rich. Thus, there are good reasons to believe that the poor should show opportunity cost neglect to a lesser extent.

To our knowledge, this idea that poor people are less susceptible to opportunity cost neglect has not yet been tested empirically. We report a series of five (quasi‐)experiments that examine whether the poor and the rich differ in how they deal with opportunity costs. In our studies, we tried to stay as close as possible to existing research on both opportunity cost neglect and research on the effects of poverty on decision making, in order to ensure comparability of our findings with the published research. The studies that we conducted used an established paradigm (Frederick et al., 2009) and a population previously used in research comparing the decisions of the poor and the rich (e.g., Callan et al., 2016; Shah et al., 2015).

In contrast to what was predicted, we find that reminding participants with low incomes of opportunity costs strongly decreases willingness to buy, implying that they neglect opportunity costs when they are not reminded (cf. Frederick et al., 2009). This effect is equally strong for participants with low incomes compared with participants with higher incomes. Furthermore, the effect is robust across measures of poverty; it is found using both objective and subjective measures of poverty. These results suggest a simple and parsimonious account of consideration of opportunity costs: Both the rich and the poor show opportunity cost neglect.

Before discussing the studies and results in detail, we first explain what opportunity costs are, why they are often neglected, and why scholars have predicted that the poor should be less susceptible to opportunity cost neglect.

## Opportunity Costs

Opportunity costs reflect the potential benefits of the best non‐chosen option. For example, when buying a movie ticket for $8.50, that same $8.50 cannot be used for other purchases. In this case, the opportunity costs reflect the best alternative use of the $8.50, which could be a different product or service, but could also be simply keeping the money for later. In neoclassical economics, consumers are assumed to take opportunity costs into account when evaluating a potential purchase, requiring them to consider all possible options. Experimental research, however, suggests that people often fail to fully take into account non‐presented alternatives, resulting in opportunity cost neglect (Frederick et al., 2009; Jones et al., 1998).

Frederick et al. (2009) found that reminding participants of opportunity costs led to a lower willingness to buy a particular product, which implies that participants neglected opportunity costs unless these were explicitly pointed out. For example, when choosing between a cheaper and more expensive coffee mug, participants were less likely to purchase the more expensive mug when the description of the cheaper mug included the phrase “leaving you with an extra $6.01 in cash to spend on something else” (Frederick et al., 2009, p. 556). Frederick et al. argue that people focus on explicit and salient information (i.e. the opportunity under consideration) and tend to ignore implicitly presented information (i.e. the non‐presented options: opportunity costs). The importance of considering opportunity costs was further demonstrated by Bartels and Urminsky (2015). They found that valuing future outcomes highly is only related to decreased spending when people consider opportunity costs. In their experiments, participants who felt highly connected to their future selves or discounted the future weakly spent less only when they were reminded of opportunity costs.

Other studies provide direct or indirect evidence for the existence of opportunity cost neglect. For example, Jones et al. (1998) found that the same decision is made differently when framed as an opportunity (“Should I move to New York?”) compared with when it is framed as a choice (“Should I move to New York or stay in Chicago?”). Specifically, given that an option is perceived as at least mildly attractive, people are more likely to pursue it when it is presented as an opportunity instead of as a choice. Because people change their decisions when the option not to move is made explicit, these results support the idea that people tend to neglect opportunity costs. Other evidence comes from the observation that people tend to ignore the hidden zero in interpersonal and intertemporal choice (Handgraaf et al., 2003; Magen et al., 2008). When it was made explicit that choosing a smaller, sooner option meant that participants would receive $0 later and that choosing a larger, later option meant that they would receive $0 now, participants were more likely to choose the larger, later option. Again, participants' choices were influenced by reminders of opportunity costs, in this case making them more patient. More recent research has shown that this effect is driven specifically by reminding participants of the future consequences—receiving $0 later (Read et al., 2016). Together, this research suggests that people tend to focus on information that is described and tend to neglect information that is not described. Because in practice opportunity costs are typically left implicit, they are often neglected.

## Opportunity Cost Neglect in the Poor

People may be especially likely to ignore implicit alternatives when the decision involves low‐cost products and when decision makers have considerable “slack” in their budgets (Zauberman & Lynch, 2005). In other words, when the impact of the trade‐offs that have to be made is limited, people are more likely to ignore opportunity costs. By the same reasoning, when trade‐offs are significant (i.e. when the decision involves high‐cost products or the decision‐maker's budget lacks slack), people should weigh opportunity costs more heavily in their decisions. In those situations, we should observe less pronounced opportunity cost neglect. In the words of Frederick et al. (2009, p. 559): “very poor individuals or those on fixed incomes may be keenly aware of opportunity costs in many decisions because their binding budget constraints may frequently necessitate a careful comparison of mutually exclusive options.” The idea that the poor may be less susceptible to opportunity cost neglect fits with a broader perspective on poverty forwarded by Mullainathan and Shafir (2013), which posits that resource scarcity promotes trade‐off thinking: Pressing needs make trade‐offs (and therefore opportunity costs) highly accessible.

Diminished sensitivity to opportunity cost neglect among the poor has not yet been directly studied, even though it fits closely with research showing that the poor are less susceptible to classic context effects (Shah et al., 2015). For example, Shah et al. presented participants with Thaler's (1985) classical beer‐on‐the‐beach scenario, where participants are asked to name their maximum willingness to pay for a beer that would be consumed on the beach, but would be bought in either a fancy resort or a run‐down grocery store. Poorer participants more often mentioned trade‐offs as the main consideration in their decision, and their willingness to pay was not influenced by where the beer was bought.

In research strongly related to the current studies, Spiller (2011) found that participants were more likely to consider opportunity costs when they were made to feel budget constrained by being paid in short pay cycles. Participants encountered a sequence of products of which they could buy some but not all and had the option to consider products available in the future (i.e. they could consider opportunity costs). Those on a “weekly” instead of “monthly” pay cycle—those who faced more constraint—were more likely to look ahead. Finally, Fernbach, Kan, and Lynch (2015) found that budget constraint made people more likely to use priority planning instead of efficiency planning. As priority planning involves explicit consideration of opportunity costs, this provides additional evidence for the effect of feeling financial constraint on considering opportunity costs.

In sum, researchers have forwarded both theoretical and empirical reasons to expect that poor people are less susceptible to opportunity cost neglect. In research studying the situational effects of inducing scarcity (e.g., Spiller, 2011), participants who were manipulated to feel more budget constrained weighted opportunity costs more heavily. However, the claim that people who are structurally poor are more likely to spontaneously consider opportunity costs has not yet been tested. Being poor often involves experiencing scarcity, but it is nonetheless important to distinguish the effects of situational scarcity from the effects of structural poverty. People in poverty do not always experience budget constraint, and poverty has many other effects besides budget constraint. The finding that budget constraint reduces opportunity cost neglect does therefore not imply that people in poverty neglect opportunity costs to a lesser extent.

## Current Research

We tested whether people with low incomes show opportunity cost neglect to a lesser extent than people with higher incomes. In all experiments, participants in the control condition were simply asked to make a choice, whereas participants in the experimental condition made the same choice after being reminded of opportunity costs. It is important to note for our present focus on decision making by the poor that previous experiments using similar manipulations mostly used student samples (Frederick et al., 2009; Jones et al., 1998), which tend to be from a more privileged background than the average population (e.g., Henrich et al., 2010). For this reason, we expected to replicate Frederick et al.'s findings that reminding of opportunity costs leads to lower willingness to buy for the richer participants. However, if the poor indeed spontaneously consider opportunity costs, the poorer participants should not be influenced by this manipulation to the same extent. Thus, we hypothesized that reminding participants of opportunity costs causes a decrease in willingness to buy for the rich but not (or to a lesser extent) for the poor.

Before we turn to the experiments, we would like to note that there are many definitions of poverty. To make sure we do not miss the effect of a particular type of poverty, we test our hypotheses using multiple poverty measures. First, we use *effective income*, calculated by dividing recoded household income by the square root of the number of people in the household (cf. Buhmann & Rainwater, 1988). Second, we test for differences between people below and above the US *Federal Poverty Guideline* (Office of the Secretary, 2015) and people in the lowest income quintile versus those in other quintiles. Finally, we use two subjective measures in which we ask people to rate their own financial situation and subjective social status (*subjective wealth* and the *MacArthur ladder*). Thus, we will examine whether opportunity cost neglect is moderated by effective income, by living below the poverty line, by being in the first income quintile, and by subjective wealth and subjective social status.

## Experiments

All experiments used a similar paradigm: Participants read a scenario about encountering an attractive product and were asked whether they would buy the product. We varied between participants whether they were reminded of opportunity costs before making the decision or not. Experiments 1–4 used the same manipulation as Frederick et al.'s (2009) study 1: The non‐buying option was phrased as “not buying the product” in the control conditions and as “keeping the money for other purchases” in the experimental conditions. In experiment 5, one group of participants was asked to list what other things they would be able to buy if they would not buy the product (in this case a tablet); another group was asked to list what they would not be able to buy if they would buy the product, and the control group simply made the decision to buy the product or not (similar to Jones et al., 1998). Because the results did not differ significantly between the two experimental conditions, they are discussed together (data on all conditions is available online). In order to examine the influence of the price of the product and the nature of the product (material or experiential; Van Boven & Gilovich, 2003), we used four different products (DVD, tablet, movie ticket, and concert ticket, refer to Table 1). To further test the idea that the poor are more likely to spontaneously consider opportunity costs, after making the buying decision in experiments 3 and 4, participants were asked to list alternative things they would do with the money. In experiments 3–5, participants also rated how difficult it was to come up with alternatives (except for participants in the control condition of experiment 5). After the scenario, we asked for income and other demographic information.

**Table 1:** Descriptive statistics, *χ* ^2^ tests, and logistic regressions for experiments 1–5

	Descriptive statistics					*χ* ^2^ test condition		Logistic regressions		
Exp.	*N*	Product	Price	Percentage choosing to buy (control condition)	Percentage choosing to buy (opp. cost condition)	*χ* ^2^	*p*	Condition (0 = control, 1 = opp. costs)	Effective income (centered, in $10,000)	Condition × effective income
1	320	DVD	$14.99	43.83%	37.97%	0.904	.342	−0.24 (0.23)	−0.08 (0.08)	0.19 (0.10)
2	328	Tablet	$249.99	62.28%	53.42%	2.289	.130	−0.40 (0.23)	−0.05 (0.06)	0.26 (0.10)**
3	642	Movie ticket	$8.50	71.97%	55.18%	18.775	<.001	−0.82 (0.18)***	0.12 (0.06)	−0.12 (0.08)
4	637	Concert ticket	$50.00	60.06%	49.69%	6.502	.011	−0.45 (0.17)**	0.03 (0.05)	0.03 (0.07)
5	511	Tablet	$249.99	68.89%	40.48%	36.507	<.001	−1.16 (0.20)***	0.08 (0.07)	0.08 (0.09)

* *p* < .05.

** *p* < .01.

*** *p* < .001.

In all five experiments, we hypothesized an interaction effect between condition and income: The effect of reminding participants of opportunity costs should be smaller for participants with lower incomes than for those with higher incomes. Furthermore, we expected that participants with lower incomes think that it is easier to come up with alternative uses of the money and spend less time per generated alternative.

### Method

#### Participants

US participants were recruited online (total *N* = 2438, 54.1% male, *M*_age_ = 31.12, *SD*_age_ = 13.03) via Amazon's Mechanical Turk (MTurk, refer to Buhrmester et al., 2011; Paolacci & Chandler, 2014), limited to people who had not participated in one of the previous experiments. For the first experiment, sample size was based on Shah et al.'s (2015) study 1B, who found an interaction effect between condition and socioeconomic status on willingness to pay at *η*_p_^2^ = 0.0315. A power analysis (with *α* = 0.05, 1 − *β* = 0.80) indicated that a minimum of 244 participants would be needed to detect this effect size. For experiments 2–5, we determined the number of participants using similar power analyses based on effect sizes from the previous experiments (*N*_1_ = 320, *N*_2_ = 328, *N*_3_ = 642, *N*_4_ = 637, and *N*_5_ = 511).

To examine whether the poor and rich differ in their decisions, heterogeneity with respect to income is necessary. In our samples, there was substantial variation in the household income of the participants, although income was in general lower than the average of the US population (DeNavas‐Walt & Proctor, 2015). In terms of household income, across the five experiments, 31.90% of the participants were in the lowest income quintile of the US population, and 25.05, 23.98, 14.16, and 4.91% were in the second, third, fourth, and fifth quintiles, respectively. Approximately 15.44% of the sample fell below the US Federal Poverty Guideline (Office of the Secretary, 2015).

#### Procedure

In all experiments, participants were presented with a scenario describing an attractive product (adapted from Frederick et al., 2009). For example, experiment 1 used the following scenario:

Imagine that on your most recent visit to the video store you come across a special sale on a new DVD. This DVD is one with your favorite actor or actress, and your favorite type of movie (such as a comedy, drama, thriller, etc.). This particular video that you are considering is one you have been thinking about buying a long time. It is available at a special sale price of $14.99.

Experiments 2–5 used different products (a tablet for $249.99, a movie ticket for $8.50, and a concert ticket for $50.00) with a similar description (refer to Table 1 for an overview of the setup and results of all experiments and Supplement A for all scenarios). Next, participants in the control condition were asked whether they would buy the product or not. Participants in the opportunity cost condition in experiments 1–4 were asked whether they would buy the product or keep the $X for other purchases. In experiment 5, participants in the opportunity cost conditions were first asked (1) what other things they would buy with the $249.99 if they would not buy the tablet or (2) what other things they would not be able to buy, if they bought the tablet for $249.99. Then, they were asked whether they would buy or not buy the tablet. Participants in the control condition were simply asked whether they would buy the tablet. In experiments 3 and 4, after making the buying decision, participants were asked to list other things they would consider doing with the money instead of buying the product. We also measured the time it took them to come up with the alternatives. In experiments 3–5, participants were asked how hard they thought it was to come up with alternatives, on a scale of 1 (very easy) to 7 (very hard).

Finally, participants were asked their household's income, the number of persons in their household, and their education level, gender, and age. Household income was asked in income brackets of $10,000, with a highest category of $150,000 and above. For the analyses, income was recoded following Ravallion (1992): Income was estimated as the midpoint of each income bracket, except for the lowest bracket (80% of the upper bound) and the highest income bracket (130% of the lower bound). We also asked participants to position themselves on the MacArthur ladder (Adler & Stewart, 2007), a measure of perceived relative social status, and to rate three subjective wealth items measuring perceived personal financial situation (on scales of 1–7 with different anchors, e.g., “How would you describe your current financial situation?”; Gasiorowska, 2014).

### Results

#### Approach to the analyses

**Figure 1:**
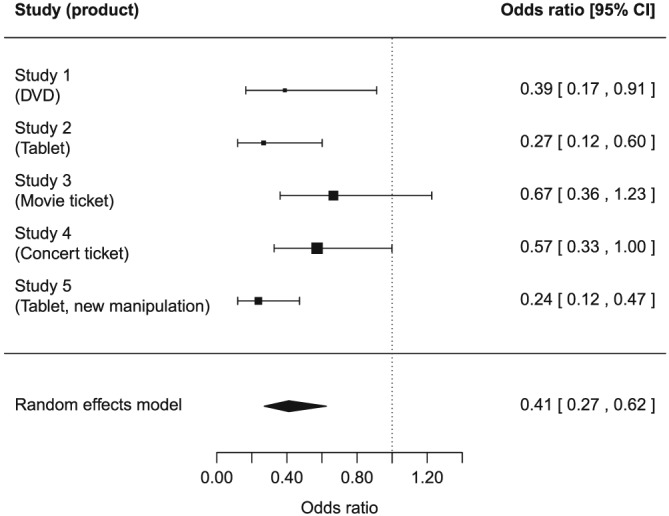
Forest plot for the meta‐analysis of the effect of condition (control vs. opportunity cost reminder) on buying decision after controlling for effective income and an interaction effect between condition and effective income for experiments 1–5. An odds ratio smaller than 1 means that participants were less likely to buy the product when the opportunity cost reminder was present than when it was not present

**Figure 2:**
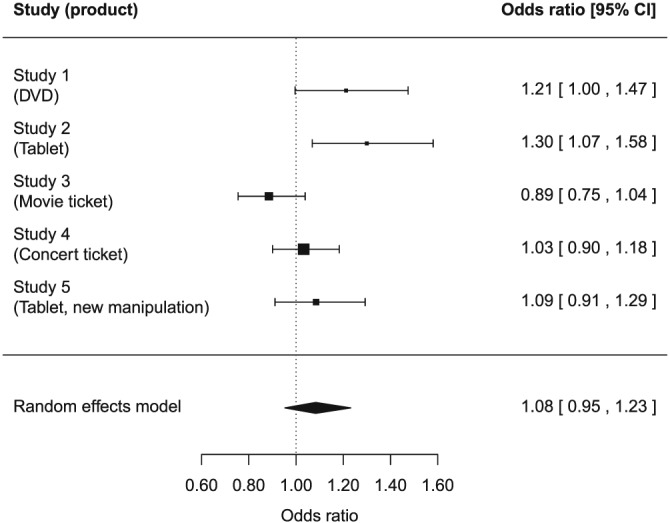
Forest plot for the meta‐analysis of the interaction effect of condition (control vs. opportunity cost reminder) and income on buying decision, after controlling for the main effects of condition and income for experiments 1–5. The coefficients represent differences in *log*(odds ratio). A positive interaction effect means that the effect of the opportunity cost reminder was larger for participants with lower incomes

For each experiment, we ran a logistic regression with buying decision as dependent variable and condition, centered effective income, and their interaction as predictors. In all analyses, condition was recoded to a dummy variable (0 = control, 1 = opportunity cost reminder). The results for each experiment are summarized in Table 1 and described in the next sections. Before testing the hypothesized interaction effects, we tested for effects of reminding of opportunity costs and income on choice; we found evidence for both these main effects. In order to test the hypotheses across the five experiments, we also report several meta‐analyses, which all use random‐effects models in the *metafor* package 1.9–9 with R 3.3.3 (R Core Team, 2016; Viechtbauer, 2010). The results of the meta‐analyses are shown in Figures 1 and 2.

#### Effect of condition

In all experiments, the proportion of participants indicating that they would buy the product was lower in the opportunity cost condition than in the control condition (the difference ranged from 5.8 to 16.8 percentage points), but this difference was significant only in experiments 3–5 (Table 1). When controlling for effective income and the interaction effect in a logistic regression, willingness to buy was still lower in the opportunity cost condition across all experiments (and statistically significant in experiments 3–5). In a meta‐analysis across the five experiments (Figure 1), the effect of condition after controlling for effective income and the interaction effect was significant and substantial, OR = 0.54, *z* = −3.81, *p* < .001, 95% CI [0.39, 0.74] (test for heterogeneity: *Q*(4) = 13.25, *p* = .010). On average, in the control conditions, 62.8% of the participants indicated buying the product, whereas only 47.8% did so in the opportunity cost conditions. In sum, we find strong evidence for opportunity cost neglect across our experiments, replicating Frederick et al. (2009).

#### Effects of income and subjective wealth

In all experiments, effective income was positively related to willingness to buy, but this correlation was only statistically significant in experiment 5 (it ranged from *r*(315) = .04, *p* = .511, 95% CI [−.07, .15] in experiment 1 to *r*(499) = .14, *p* = .002, 95% CI [.05, .22] in experiment 5). A meta‐analysis of the correlations between effective income and buying decision shows a small but statistically significant correlation, *r* = .07, *z* = 3.59, *p* = <.001, 95% CI [.03, .11] (*Q*(4) = 2.82, *p* = .588). In another meta‐analysis, subjective wealth was also positively related to buying decision, *r* = .13, *z* = 6.41, *p* < .001, 95% CI [.09, .17] (*Q*(4) = 2.99, *p* = .560).

#### Interaction condition and income

Contrary to the hypothesis that the poor show opportunity cost neglect to a lesser extent than the rich, none of the studies showed a statistically significant negative interaction effect between effective income and condition (Table 1). Experiment 2 even found a significant positive interaction effect, *b* = 0.26, *t*(317) = 2.62, *p* = .009, meaning that reminding participants of opportunity costs led to a greater decrease in willingness to buy for participants with lower incomes than for participants with higher incomes, which is opposite to what was predicted. In a meta‐analysis, we also found no significant interaction effect between condition and effective income, *b* = 0.08, *z* = 1.22, *p* = .222, 95% CI [−0.05, 0.21] (*Q*(4) = 11.03, *p* = .026; effective income in $10,000, refer to Figure 2). The natural logarithm of this coefficient, ln(*b*) = 1.08, 95% CI [0.95, 1.23]) indicates the ratio of the odds ratios in the sample for the different conditions. The lower bound of its 95% confidence interval is close to 1, which means that the data suggest that even if a negative interaction effect exists, it would be small. Furthermore, the data show more support for a positive interaction effect; in the sample, the effect of opportunity costs was bigger (more negative) for participants with lower incomes.

As a robustness check, we also tested whether the effect of condition on buying decision was moderated by any of the other wealth measures. In logistic regressions with subjective wealth instead of effective income, none of the interaction effects were statistically significant (*p*‐values ranged from .139 to .724). A meta‐analysis on these interaction effects also showed no significant effect, *b* = 0.11, *z* = 1.38, *p* = .169, 95% CI [−0.04, 0.26] (*Q*(4) = 5.23, *p* = .264). Similarly, using the MacArthur ladder in a similar meta‐analysis also yielded no significant interaction effect, *b* = 0.05, *z* = 0.64, *p* = .522, 95% CI [−0.11, 0.21] (*Q*(4) = 9.62, *p* = .047), as did meta‐analyses using five effective income brackets (i.e. equally sized groups of participants divided by effective income), *b* = 0.09, *z* = 0.79, *p* = .431, 95% CI [−0.13, 0.31] (*Q*(4) = 12.35, *p* = .015) or analyses that divided participants into those in the first income bracket versus other income brackets, *b* = 0.18, *z* = 0.78, *p* = .434, 95% CI [−0.27, 0.62] (*Q*(4) = 5.46, *p* = .243), first income quintile versus other quintiles, *b* = 0.41, *z* = 1.85, *p* = .064, 95% CI [−0.02, 0.84] (*Q*(4) = 5.39, *p* = .250), or below the poverty line versus above the poverty line, *b* = 0.05, *z* = 0.19, *p* = .848, 95% CI [−0.43, 0.53] (*Q*(4) = 4.95, *p* = .293).

Because there was significant heterogeneity in the effect size of the interaction effect between condition and effective income on buying decision, we conducted some exploratory analyses to test whether the size of this effect was moderated by any study‐level moderators (Supplement B). The effect was not significantly moderated by the price of the product nor the manipulation used. However, the interaction effect was significantly more positive for material than for experiential products. Furthermore, a meta‐analysis on only the experiments with material products found a significant positive interaction effect. This means that, for material products, the effect of reminding of opportunity costs was stronger (more negative) for the poor than for the rich. Note that this effect is opposite to that we had hypothesized.

In sum, these results do not support the prediction that the poor show less or no opportunity cost neglect. Overall, the data indicate that the poor are as likely as the rich to fail to take opportunity costs into account. If anything, our exploratory analyses indicate that under some conditions, the poor may be *more* likely than the rich to show opportunity cost neglect.

#### Generating alternatives

If the poor are more likely to spontaneously consider opportunity costs, they should find it easier to generate alternative uses of the money. To test that, participants in experiments 3–5 were asked to list alternative ways to use the money. Participants with lower incomes reported that they found it easier to come up with alternatives, although the correlation between effective income and perceived difficulty was only statistically significant in experiment 3, *r*(594) = .09, *p* = .035, 95% CI [.01, .17]. In a meta‐analysis across the three experiments, the correlation was small but statistically significant, *r* = .07, *z* = 2.93, *p* = .003, 95% CI [.02, .12] (*Q*(2) = 0.32, *p* = .851).

The number of generated alternatives correlated positively with income in experiment 4, *r*(582) = .11, *p* = .006, 95% CI [.03, .19] and non‐significantly in experiment 3, *r*(594) = .05, *p* = .244, 95% CI [−.03, .13], and experiment 5, *r*(499) = .07, *p* = .120, 95% CI [−.02, .16]. A meta‐analysis on these correlations showed a small but significant positive correlation between effective income and the number of alternatives generated, *r* = .08, z = 3.33, *p* < .001, 95% CI [.03, .12] (*Q*(2) = 1.50, *p* = .472). After controlling for the number of alternatives generated, there was no statistically significant effect of effective income on time spent per generated alternative in any of experiments 3–5 (*p* values ranged from .313 to .928), nor in a meta‐analysis across the three experiments, OR = 0.74, *z* = −1.03, *p* = .302, 95% CI [0.41, 1.32] (*Q*(2) = 0.70, *p* = .706). 1As described in the Supporting Information, we also coded the alternatives on whether they were material versus experiential products and necessities versus luxurious products. We found no effect of income on listing material versus experiential products. Participants with higher incomes were somewhat more likely to list luxurious products rather than necessities compared with participants with lower incomes.


In sum, there is no clear evidence that alternatives come more easily to mind for participants with lower incomes. We find some evidence that people with lower incomes find it easier to come up wither alternative uses of their money. However, people with lower incomes generated *fewer* alternatives than participants with higher incomes. We found no differences in the time spent per generated alternative, after controlling for the number of generated alternatives.

## General Discussion

Across five experiments, we replicate the finding by Frederick et al. (2009) that reminding people of opportunity costs decreases willingness to buy. However, this effect was equally strong for participants with low and high incomes: Both showed a decrease in willingness to buy in response to the reminder. In other words, we found no evidence for an interaction effect between income and condition: Both the rich and the poor showed opportunity cost neglect.

These findings contribute additional evidence for the robustness of opportunity cost neglect as described by Frederick et al. (2009). People appear to fail to fully consider opportunity costs in buying decisions. The findings contradict the idea, proposed by several authors (Frederick et al., 2009; Mullainathan & Shafir, 2013; Shah et al., 2015; Spiller, 2011), that opportunity costs are more salient for the poor. Although previous research did find an effect of budget constraint on opportunity cost consideration (Spiller, 2011), we did not find evidence of a similar effect of poverty. This may imply that the poor do not continuously experience resource scarcity, even though they may be more likely to encounter situations of scarcity. Our data suggest that people only think about opportunity costs when they are relevant or salient.

One potential alternative explanation for our findings could be that the poorest participants in our studies were not poor enough. We do not consider this a viable explanation. In line with previous work on MTurk samples (Buhrmester et al., 2011; Paolacci & Chandler, 2014), our samples display substantial variation in income. On average, about 15.44% of the participants in our experiments lived below the US Federal Poverty Guideline (Office of the Secretary, 2015). This is corroborated by the fact that both effective income and subjective wealth did affect willingness to buy in a meta‐analysis across the experiments, suggesting at least a substantial amount of variance in income. Furthermore, studies by Shah et al. (2015) used MTurk samples in similar paradigms and did find interaction effects between experimental condition and income on financial decisions. Therefore, we do not think that our findings can be explained by inadequate sampling. Of course, we cannot fully exclude the possibility that people living in extreme poverty would not show opportunity cost neglect. However, even if this were true, this would confine the idea that opportunity costs are more salient for the poor to a very small subsample of the total population of people generally seen as “poor” in studies of poverty and decision making.

We tested only a restricted set of poverty measures and products. For instance, we did not include a measure of childhood socioeconomic status, which only modestly correlates with current socioeconomic status but also impacts financial decisions made later in life (Griskevicius et al., 2013). 2We thank an anonymous reviewer for suggesting this alternative explanation. Future research could test whether growing up in a budget‐constrained environment leads to less opportunity cost neglect later in life. We also only used scenarios with hedonic, as opposed to utilitarian, products. We chose these products because opportunity costs should be higher for hedonic than for utilitarian products. When thinking about whether or not to buy a movie ticket, it is more likely that there are useful alternative uses of the money than when thinking about spending money on groceries. Especially for people with low incomes, opportunity costs should be more pressing and therefore come to mind more easily for hedonic goods. Therefore, we feel that this is the strongest test of the hypothesis.

It is also possible that the hypothesized difference was not found because participants did not think deeply about their decision, because the decisions were hypothetical. Again, we do not think this to be a likely explanation, because it is hard to reconcile with the effects of income on hypothetical choices in studies by Shah et al. (2015). In addition, this explanation has trouble explaining why willingness to buy would be influenced by effective income (although this effect was small) and by reminding of opportunity costs. If participants are not thinking deeply or not paying attention, their decisions should not be influenced by any of these variables. Finally, Frederick et al. (2009) replicated the effect of reminding of opportunity costs on willingness to buy in a study using consequential choices, suggesting that people behave similarly when the choices are consequential.

Another alternative explanation is that the reminder of opportunity costs had no effect on most of the poor participants, but a strong effect on some. In other words, whereas most poor participants were already considering opportunity costs, the reminder was particularly effective for the minority who were not. Under the assumption that people who spontaneously consider opportunity costs find it easier to generate alternative uses for their money, we can test this explanation by examining whether there is a three‐way interaction between condition, income, and reported difficulty of generating alternatives: The effect of the opportunity cost reminder should interact with the difficulty variable for people with low incomes, but not for those with high incomes. We do not find such an effect in experiments 3 and 4 (*p* values >.230), nor in a meta‐analysis across the two studies, *b* = 0.03, *z* = 0.94, *p* = .347, 95% CI [−0.04, 0.10] (*Q*(1) = 0.57, *p* = .451).

A further possibility is that the poor are more likely to think about opportunity costs, but not more likely to act on them. The opportunity cost reminder might exert more normative pressure for participants with lower incomes than for participants with higher incomes. However, we think that this is a less parsimonious explanation of our data, and we have no reason to believe that for lower income individuals, the link between cognition and behavior is weaker. Furthermore, we think that it is unlikely that the fairly subtle reminder of opportunity costs exerts a strong normative pressure.

Finally, it is possible that a third variable, intelligence, has an effect on both income and the consideration of opportunity costs, negating the effect of income. However, even if intelligence was to affect both income and opportunity cost neglect, our data would still contradict the claim that the poor are more likely to think about opportunity costs. Furthermore, we do not find strong evidence of an effect of education in our studies; a meta‐analysis of the effect of condition and education and their interaction on buying decision showed no significant interaction effect, OR = 1.13, *z* = 1.91, *p* = .056, 95% CI [1.00, 1.27] (*Q*(4) = 4.78, *p* = .311). When education was added to the meta‐analysis of the effect of condition, effective income, and their interaction on buying decision, the interaction effect was still not significant, OR = 1.08, *z* = 1.25, *p* = .211, 95% CI [0.96, 1.23] (*Q*(4) = 10.62, *p* = .031).

Our findings propose a number of suggestions for future studies and policy. First, the difference between our findings and those by Spiller (2011), who found that opportunity cost neglect was affected by budget constraint, raises questions about the different effects of structural poverty and situational budget constraint on financial decision making. Studies on poverty and decision making typically make use of either quasi‐experimental designs using existing groups of people who live under different conditions or of experimental designs using situational inductions of scarcity. Our findings and those by Spiller suggest that the results obtained with one design do not necessarily generalize to other designs. It would be interesting to see whether similar differences occur with other dependent variables studied in a context of scarcity or poverty. For example, the poor are less likely to be affected by a decision's context (Shah et al., 2015), but does experiencing scarcity also reduce the impact of context? Second, our exploratory analyses suggest that there might be a difference between material products and experiences: We found that the opportunity cost reminder had a stronger effect on low‐income individuals than high‐income individuals for material but not for experiential products. Previous research found that facing financial constraints increases interest in material over experiential products (Tully et al., 2015). Possibly, this change in preferences is associated with more opportunity cost neglect for material products. Third, the replication of findings by Frederick et al. (2009) in a socioeconomically diverse sample suggests that a simple reminder of opportunity costs might be a useful way to help both poor and rich consumers make choices that are more in line with their long‐term goals. Finally, the finding of evidence for opportunity cost neglect in the poor may mean that more attention should be paid to this factor when trying to alleviate poverty. After all, neglecting opportunity costs might have more harmful consequences for the poor because of their narrow margins of error (Bertrand et al., 2006).

To conclude, our data suggests that poor and rich alike are susceptible to opportunity cost neglect. Opportunity costs do not seem to be on the top of the minds of people, regardless of their income. These findings are unlikely to be explained by sampling, methodology, or unobserved variables. Thus, the most parsimonious interpretation is that opportunity cost neglect is a robust and general phenomenon.

## Supporting information

Table S1. Categories of coded alternatives, experiments 3 and 5
