# Supplementary material for: Evidence for Opportunity Cost Neglect in the Poor
Source: J Behav Decis Mak. 2017 Sep 11;31(1):65–73. doi: 10.1002/bdm.2041 (PMC5763356; doi:10.1002/bdm.2041)
Supplement: Supplementary file 1 — Table S1. Categories of coded alternatives, experiments 3 and 5 [file BDM-31-65-s001.docx]

# Supplement A

Scenarios used in the experiments

These are the different scenarios presented to participants in all experiments. For Experiments 1–4, the only change across conditions was the wording of the second option: either “Not buy this [DVD]” or “Keep the [$14.99] for other purchases”. For experiment 5, participants were asked (1) what other things they would buy with the $249.99 if they would not buy the tablet, (2) what other things they would not be able to buy, if they bought the tablet for $249.99, or (3) not asked anything, and then made the buying decision.

**Experiment 1**

Imagine that on your most recent visit to the video store you come across a special sale on a new DVD. This DVD is one with your favorite actor or actress, and your favorite type of movie (such as a comedy, drama, thriller, etc.). This particular video that you are considering is one you have been thinking about buying a long time. It is available at a special sale price of $14.99.

What would you do in this situation?

- Buy this DVD
- Not buy this DVD [Keep the $14.99 for other purchases]

**Experiment 2**

Imagine that you have been saving some extra money on the side to make some purchases, and on your most recent visit to the mall you come across a special sale on a tablet. This tablet is one of your favorite brand with good specifications. This particular tablet that you are considering is one you have been thinking about buying a long time. It is available at a special sale price of $249.99.

What would you do in this situation?

- Buy this tablet
- Not buy this tablet [Keep the $249.99 for other purchases]

**Experiment 3**

Imagine that a new movie came out that is showing tonight. This movie is your preferred genre, with your favorite actor/actress. A movie ticket costs $8.50.

What would you do in this situation?

- Buy a movie ticket
- Not buy a movie ticket [Keep the $8.50 for other purchases]

**Experiment 4**

Imagine: Tonight a concert is scheduled close to where you live. You have been thinking about going to this concert for a long time. Tickets are available at $50.

What would you do in this situation?

- Buy a concert ticket
- Not buy a concert ticket [Keep the $50 for other purchases]

## Experiment 5

Imagine that you have been saving some extra money on the side to make some purchases, and on your most recent visit to the mall you come across a special sale on a tablet. This tablet is one of your favorite brand with good specifications. This particular tablet that you are considering is one you have been thinking about buying a long time. It is available at a special sale price of $249.99.

[What other things would you buy with the $249.99 if you wouldn't buy the tablet? Please list the things you would consider buying with the money below. You can list multiple things, use a separate line for each thing.]

[If you would buy the tablet for $249.99, what other things would you not be able to buy? Please list the things you would not be able to buy below. You can list multiple things, use a separate line for each thing.]

What would you do in this situation?

- Buy this tablet
- Not buy this tablet

# Supplement B

Exploratory analyses

## Meta-regression interaction condition and income

Because there was significant heterogeneity in the effect sizes of the interaction effects across the studies (τ^2^ = 0.014, *Q*(4) = 11.03, *p* = .026), we conducted an exploratory meta-regression and several meta-analyses to test for potential study-level moderators. Specifically, we regressed the interaction effect between condition and effective income on buying decision on the potential moderators product price (high vs. low), product type (material vs. experiential), and the manipulation used (i.e., the paradigm used by Frederick et al., 2009 versus the new manipulation in Experiment 5). The interaction effect was not significantly moderated by the price of the product, *OR* = 1.13, *z* = 1.44, *p* = .150, 95% CI [0.96, 1.34], nor the manipulation used, *OR* = 0.81, *z* = -1.70, *p* = .089, 95% CI [0.64, 1.03]. However, the interaction effect was significantly more positive for material than for experiential products, *OR* = 1.31, *z* = 3.05, *p* = .002, 95% CI [0.10, 0.44]. In an exploratory meta-analysis with only the experiential products, the interaction effect was not significant, *OR* = 0.96, *z* = -0.51, *p* = .610, 95% CI [0.83, 1.12]. However, a meta-analysis on only the experiments with material products found a significant positive interaction effect, *OR* = 1.19, *z* = 3.09, *p* = .002, 95% CI [1.06, 1.32], which means that the effect of reminding of opportunity costs for material products was stronger (more negative) for the poor than the rich.

## Coding of alternatives

In a further exploration of the data, the alternatives participants listed in Experiment 3 and 5 were rated as necessity versus luxury and as material versus experiential (on scales of 1–9), and coded into categories. The alternatives participants listed in were coded and rated as follows: First, the responses were shortened to one or a few words. Then, these items were recoded into overarching categories. As the number of categories was still large, these categories were again recoded into broader categories, leaving 17 different categories (see Table S1). A second coder then assigned all listed alternatives to these categories, and rated whether a listed alternative could be classified as necessity versus luxury, and as material versus experiential (on scales of 1–9).

In general, participants mostly mentioned considering buying food and groceries, (16.8% of all named alternatives), entertainment products (14.2%), clothing (10.9%), or using it for transportation (e.g., buying gas, 8.0%). Effective income did not affect whether participants generated more products rated as luxuries versus necessities in Experiment 3, *r*(554) = 0.00, *p* = .922, 95% CI [-0.08, 0.09], but did in Experiment 5, *r*(318) = .18, *p* < .001, 95% CI [0.08, 0.29]. A meta-analysis across these two studies shows no significant correlation between effective income and listing items rated as luxuries, *r* = .09, *z* = 1.04, *p* = .299, 95% CI [-0.08, 0.27]. Effective income did not affect whether participants generated more products rated as experiential versus material products in Experiment 3, *r*(555) = -.01, *p* = .731, 95% CI [-0.10, 0.07], but did in Experiment 5, *r*(318) = .13, *p* = .022, 95% CI [0.02, 0.23]. In a meta-analysis, the correlation was not significant, *r* = .06, *z* = 0.78, *p* = .436, 95% CI [-0.08, 0.20]. Thus, poorer participants did not seem to think of more material or experiential products, but there is some evidence that they are more likely to think of more necessities over luxurious products.

Table S1

*Categories of coded alternatives, Experiment 3 and 5*

| Category | Frequency | Percentage of total responses |
| --- | --- | --- |
| food/groceries | 489 | 16.8% |
| entertainment products | 414 | 14.2% |
| clothing | 318 | 10.9% |
| transportation | 232 | 8.0% |
| go out to eat | 209 | 7.2% |
| saving | 203 | 7.0% |
| household | 171 | 5.9% |
| bills | 153 | 5.3% |
| rent or buy a movie/online subscription | 133 | 4.6% |
| activity/hobby | 127 | 4.4% |
| alcohol/tobacco | 120 | 4.1% |
| miscellaneous | 119 | 4.1% |
| snacks | 106 | 3.6% |
| gift/donation | 58 | 2.0% |
| pay off debt | 34 | 1.2% |
| none | 26 | 0.9% |
